# Royal South Hants Infirmary, Southampton

**Published:** 1892-07-23

**Authors:** 


### THE INSTITUTIONAL WORKSHOP.

### WITHIN THE HOSPITALS.

### VI.—ROYAL SOUTH HANTS INFIRMARY, SOUTHAMPTON.

This institution, which has been in existence for fifty-three years, contains 104 beds, the average number occupied being about eighty. A good part of the present buildings are old and out of date, and although efforts have been made to improve the sanitary condition of this portion of the institution, and in various ways to bring it more up to modern requirements, it is desirable, for the credit of Southampton, that much of it should be pulled down and rebuilt. This might be done by demolishing the wards on either side of the old administration block, leaving the latter standing. It would be well at the same time to pull down and remove the numerous ramshackle outbuildings, which are unsightly, out of date, and largely useless. The centre building was erected, we believe, in 1838, and at that time the site appears to have been free from contiguous buildings; but, subsequently, a chapel has been put up, which has rendered one side of the hospital almost unfit for ward purposes. It would be interesting to know how the Committee could have allowed the erection of this chapel, seeing that its buildings damage the ward accommodation, and tend seriously to interfere with the treatment and well-being of the patients admitted to these wards. In any case, having regard to the important position which Southampton occupies among southern towns, and its rapid development as an important port, we would earnestly urge the Committee to summon a towns-meeting, having first selected a capable architect and prepared plans for the reconstruction and remodelling of the existing buildings. Portions of the infirmary, *i.e.*, the wards, which are known, we believe, as the Eyre Crabbe Wing, are satisfactory. The present buildings consist of two distinct parts, one good and one bad. The latter should be gutted, new wards erected on the site, which might easily be done, and the whole of the sanitary arrangements remodelled. We feel sure that the serious defects of the present buildings have only to be pointed out and brought home to the consciences of the people of Southampton to secure an abundance of funds to enable the committee to reconstruct the older portions of the infirmary. An urgent appeal should be promptly issued so as to enable the committee to secure that the medical and surgical work of the infirmary shall be conducted under the most favourable conditions which modern science and experience can suggest. The utter inadequacy of the older building must be patent to everybody, and it is essential to the credit of Southampton that this noble foundation shall be promptly made to comply with the advanced rules of hospital construction.

#### THE OUT-PATIENT DEPARTMENT.

Like all the older institutions, until quite recently the out-patient department was situated in the basement, and so long as that was the case the wards must have been in anything but a sanitary condition. Recently a new out-patient department has been erected at a cost of £4,000, which sum was raised to commemorate Her Majesty's Jubilee. The building consists of a central hall, with physicians' and surgeons' rooms and a dispensary. The system of out-patient registration seems to be well suited to the purpose. The fact that this wing has been erected affords evidence of the desire of the committee to secure the utmost efficiency within their power, and we have some confidence therefore that they will lose no time in forming a Building Committee, and set to work to raise the necessary funds to reconstruct the main building. In the grounds a wooden building has been erected, called a fever cottage, but its condition presents such evidence of neglect and unsuitability that we are sure the medical staff would not feel justified in using it for the treatment of patients. In these circumstances it would be well to pull it down, and to make arrangements with the municipal authorities for the treatment of all such cases in a municipal infectious hospital, under the supervision of the Medical Officer of Health.

#### GOOD POINTS.

As we have already stated, the wards in the Eyre Crabbe Wing are good. The whole institution presented a very clean and well-kept appearance, and reflects credit upon the management. Like most of the southern hospitals these wards have excellent verandahs at one end, into which it is possible to wheel the patients in fine weather. The importance of these verandahs has been proved to demonstration in the German hospitals, and we imagine they must be found by the surgeons of great importance in the treatment of serious cases. The nursing appears to be good. Several of the nurses were trained at Winchester, and those we spoke to not only showed a loyal attachment to their *alma mater*, but gave evidence of skilful training and marked efficiency. The uniforms were becoming and effective, and the appearance of the wards, especially in the Eyre Crabbe Wing, left little to be desired. Formerly the accommodation for the nurses was very bad, but during the year 1891 some cottages belonging to the infirmary have been converted into the nurses' home, which contains a most attractive sitting-room, and so the nursing staff is now comfortably provided for. We hope, however, that in any reconstruction that may take place the committee will decide to substitute Rufford's baths for the painted metal ones at present in use. At this institution probationers are received and trained as rural nurses. They remain for six months, and are then passed on to the lying-in hospital to be trained in midwifery and subsequently go through a course of district nursing so as to fit them for their duties. Great care seems to be exercised in selecting suitable women for this work, which we hope will be greatly extended, as the services of trained nurses are badly needed in many portions of rural England.

#### THE MEDICAL STAFF.

This consists of two physicians and three surgeons, with an assistant-physician, an assistant surgeon, and a dentist. There is also a house-surgeon, Dr. H. P. Ward, to whom Mr. F. W. Bartlett acts as assistant. The resident medical officers remain in the service of the infirmary for some few years, to the no small advantage of the patients and the institution generally. The committee are to be congratulated in having so intelligent and able a house-surgeon as Dr. Ward most certainly is, and we were much struck with the thorough grasp which he undoubtedly possesses, not only of hospital administration in the abstract, but of the requirements and needs of the institution of which he at present has the responsible medical charge.

#### SPECIAL FEATURES.

It is a matter of congratulation that the inhabitants of the towns in the south of England where there are hospitals, take a special interest in the well-being and progress of these institutions. Thus certain ladies devote a good deal of time to the collection of funds, and the report shows that a considerable income is derived from the efforts of those ladies who have charge of collecting boxes, one of which produced nearly £50 during 1891. In the children's ward are several special cots, which have been presented and are maintained in memoriam, or by the special efforts of ladies who undertake the collection each year of a sum sufficient to maintain them. Thus there is the Lyndhurst cot, the Eyre Crabbe cot, the Mothers' cot, the Hardwick cot, the Lymington cot, and others. These facts afford evidence of the genuine interest which is taken in the well-being of the Royal South Hants Infirmary, and they ought to encourage the Managers to organise a special building committee, and to set to work to collect some £10,000 to enable them to reconstruct the infirmary, and to make it replete with every modern improvement. They have already the nucleus of a building fund in Sir Thomas Henry's legacy of £2,000, which is left on condition that it is expended on the erection of a new wing for the treatment of in-patients from Freemantle and its neighbourhood. The institution possesses a nurses' superannuation fund, which amounts to £629. We would strongly urge the Committee to communicate with the Managers of the Royal National Pension Fund for Nurses, with the view to the affiliation of the infirmary with that Fund upon terms which would secure upon a thrift basis an adequate pension for the whole of its nursing staff. The Committee receive less than four per cent. on their money at the present time, and much more favourable terms could be secured by affiliation with the Royal National Pension Fund.

